# Alternating Hemiplegia and Cardiac Dysrhythmia

**DOI:** 10.15844/pedneurbriefs-29-9-6

**Published:** 2015-09

**Authors:** J. Gordon Millichap

**Affiliations:** 1 Division of Neurology, Ann & Robert H. Lurie Children’s Hospital of Chicago, Chicago, IL; 2 Departments of Pediatrics and Neurology, Northwestern University Feinberg School of Medicine, Chicago, IL

**Keywords:** Alternating Hemiplegia of Childhood, ATP1A3, SUDEP, Electrocardiogram

## Abstract

Investigators at the National Hospital for Neurology and Neurosurgery, Queen Square, London, and multiple centers in the UK, Europe, US, Melbourne, Australia, and Canada, analyzed ECG recordings of 52 patients with alternating hemiplegia from 9 countries; all had whole-exome, whole-genome, or direct Sanger sequencing of ATP1A3; 47 had a confirmed missense mutation in ATP1A3.

Investigators at the National Hospital for Neurology and Neurosurgery, Queen Square, London, and multiple centers in the UK, Europe, US, Melbourne, Australia, and Canada, analyzed ECG recordings of 52 patients with alternating hemiplegia from 9 countries; all had whole-exome, whole-genome, or direct Sanger sequencing of ATP1A3; 47 had a confirmed missense mutation in ATP1A3. De novo mutation in ATP1A3 is the underlying cause of most cases. Autonomic dysfunction, cardiac symptoms, medication, and family history of cardiac disease or sudden death were recorded. Thirty-two patients were under 16 years of age; 26 were female. Three-quarters had a diagnosis of epilepsy; EEGs were not reported. Half the cohort (26/52) had resting 12-lead electrocardiogram (ECG) abnormalities; 25/26 had repolarization (T wave) abnormalities. These abnormalities were significantly more common in people with alternating hemiplegia than in an age-matched control group of 52 people with epilepsy. The average corrected QT interval was significantly shorter in people with alternating hemiplegia than in the disease control group. J wave or J-point changes were seen in 6 patients with alternating hemiplegia. Over half the affected cohort (28/52) had intraventricular conduction delay, or incomplete right bundle branch block, a much higher proportion than in the normal population or disease control cohort (P=0.0164). Abnormalities in alternating hemiplegia were more common in those >16 years old, compared with those <16 (P=0.0095). ECG changes occurred independently of seizures or plegic episodes. ECG abnormalities are common in alternating hemiplegia, with characteristics reflecting inherited cardiac channelopathies and impaired repolarization reserve. Cardiac dysfunction may account for unexplained premature mortality of patients with alternating hemiplegia. [1]

COMMENTARY. This study provides a more complete understanding of alternating hemiplegia and its relation to cardiac dysfunction. QT intervals are significantly shorter in alternating hemiplegia patients compared to controls with epilepsy. QT prolongation is reported in individuals with epilepsy, suggesting that the association of alternating hemiplegia with cardiac dysfunction and the change in QT interval are the opposite of that occurring in persons with epilepsy. That alternating hemiplegia is a form of epilepsy is suggested by the frequency of occurrence of seizures and a diagnosis of epilepsy in 40 cases (75%). Electroencephalographic (EEG) confirmation of cases in the present study is not provided. Migraine is also frequently associated and is considered as a cause; flunarizine and topiramate may prevent recurrence whereas anticonvulsants are of no benefit.

To determine the evolution of epileptic seizures in alternating hemiplegia of childhood, Saito Y and associates reviewed clinical findings of 9 patients [2]. Paroxysmal abnormal ocular movements, head turning, and tonic, clonic, or myoclonic limb movements were initial symptoms (birth-8 months) in each patient. Ictal EEG of these episodes and of accompanying hemiplegic periods later in infancy showed generalized slowing. Presumptive epileptic seizures appeared at 2-16 years in 7 patients; ictal EEGs revealed focal slow or fast activities during facial or limb twitching, and sharp waves or polyspike-wave activities during clonic/myoclonic seizures. Status epilepticus in alternating hemiplegia is linked to severe outcome with psychomotor deterioration. The variations in phenotypes may imply multiple causative genes for alternating hemiplegia [2]. A KCNQ1 mutation is recently reported in a family suffering both epilepsy and prolonged QT interval [3].
